# BEX1 is an RNA-dependent mediator of cardiomyopathy

**DOI:** 10.1038/s41467-017-02005-1

**Published:** 2017-11-30

**Authors:** Federica Accornero, Tobias G. Schips, Jennifer M. Petrosino, Shan-Qing Gu, Onur Kanisicak, Jop H. van Berlo, Jeffery D. Molkentin

**Affiliations:** 1 Cincinnati Children’s Hospital Medical Center, Cincinnati, OH 45229 USA; 2 Dorothy M. Davis Heart and Lung Research Institute, Department of Physiology and Cell Biology, Ohio State University, Columbus, OH 43210 USA; 3 Department of Medicine, Lillehei Heart Institute, University of Minnesota, Minneapolis, MN 55455 USA; 4 Howard Hughes Medical Institute, Cincinnati, OH 45229 USA

## Abstract

Regulation of mRNA splicing, processing and stability is increasingly recognized as a critical control point in dynamically altering gene expression during stress or disease. Very little is understood of this process in heart failure. Here, we show that BEX1 is a heart failure-induced gene functioning as an mRNA-associated protein that enhances expression of a subset of cardiac disease-promoting genes. Modeling the increase in BEX1 that occurs in disease, cardiac-specific BEX1 transgenic mice show worse cardiac disease with stress stimulation, whereas *Bex1* gene-deleted mice are protected from heart failure-promoting insults. Proteomic and interactive screening assays show that BEX1 is part of a large ribonucleoprotein processing complex involved in regulating proinflammatory mRNA expression in the heart. Specifically, induction of BEX1 augments the stability and expression of AU-rich element containing mRNAs typically found within proinflammatory genes. Thus, BEX1 functions as an mRNA-dependent effector that augments pathology-promoting gene expression during heart failure.

## Abstract (web summary)

Little is known about the changes in mRNA splicing, processing and stability that can alter gene expression during heart failure. Here, the authors show that BEX1 is induced during heart failure and is part of a ribonucleoprotein complex enhancing the expression and stability of proinflammatory genes.

## Introduction

Heart failure is a complex and progressive disease that evolves from an initial impairment of cardiac muscle function^1^. Current treatments are largely based on drugs that reduce neuroendocrine signalling and partially restore vascular and circulatory physiology, although patients still ultimately perish from the disease^1^. Augmented neuroendocrine signalling in heart failure is part of a larger proinflammatory response that can also be targeted with selective pharmacologic agents^1, 2^. Indeed, both innate and adaptive immune responses are activated in the heart in response to tissue injury, and many of the biological effects of proinflammatory cytokines drives heart failure in experimental animal models and in humans^3–7^. Molecular effectors underlying the innate immune response have also been implicated in cardiac inflammation and disease. For example, damage-associated molecular patterns (DAMPs) are increased in heart failure with ongoing tissue injury and initiate signalling cascades that activate nuclear factor-kappa B subunit (NF-κB) and interferon regulatory factor (IRFs) transcription factors, leading to production of proinflammatory cytokines and interferon signalling in the heart^3^. Targeting innate immunity pathways to reduce the overall burden of inflammatory signalling in the heart during failure would be an attractive therapeutic strategy.

Although heart failure is accompanied and even driven by the expression of maladaptive and proinflammatory proteins, how the synthesis of these proteins is achieved remains an area of ongoing investigation. In particular, the importance of regulating the maturation, localization and stability of mRNA is emerging as a fundamental mechanism for the control of protein synthesis during adaptive and pathological stresses^8^. From their biogenesis at the site of transcription, mRNAs associate with RNA-binding proteins (RBPs) to form ribonucleoprotein complexes^8^. There is a remarkable diversity within the 100 s of RBPs encoded in the vertebrate genome that gives rise to an intricate array of unique ribonucleoprotein complexes for selected classes of mRNAs^9, 10^.

In the heart, control of mRNA splicing has been implicated in dilated cardiomyopathy, and an increasing number of splicing factors have been suggested to underlie stress-induced pathologic cardiac remodeling and dysfunction^11–15^. Indeed, Wang and colleagues recently showed how the RNA splicing regulator RBFox1 directly impacts hypertrophy and pathology of the heart, in part by altering the differential splicing characteristics of the myocyte enhancer factor-2 gene family^11^. The stability of mRNA has also emerged as a key step in the regulation of eukaryotic gene expression. For example, RNA-stabilizing proteins can determine transcript levels by binding specific elements in the 3′ untranslated region (UTR) of subsets of target mRNAs^16, 17^. Oncogenes and cytokines represent sub-classes of mRNAs that contain specific destabilizing adenylate-uridylate-rich elements in their 3′-UTR^18, 19^. Although the function of RNA-stabilizing proteins has been widely studied in cancer biology, their role in cardiovascular stress and disease is only beginning to be understood^16^.

Here we discovered that brain-expressed X-linked protein 1 (BEX1) is a novel factor induced during heart failure, which orchestrates inflammatory-signalling through a novel RNA-dependent processing complex. BEX1 belongs to the BEX gene family, which is composed by five members of proteins with unclear function^20^. BEX1 was initially identified as a differentially expressed gene in teratocarcinoma cells stimulated to differentiate with retinoic-acid^21^. Since then, multiple groups have reported changes in expression of BEX1 in cancer^22–24^. In addition to an association with cell cycle regulation and cancer biology, previous studies suggested a role for BEX1 in skeletal muscle and neuron differentiation, as well as a role as in downstream nerve growth factor (NGF) signalling^23, 25–29^. However, the contribution of BEX1 in cardiac pathophysiology has never been addressed, nor has the molecular function of BEX proteins been annotated. Here we show that cardiac-specific BEX1 transgenic mice have worse cardiac disease with stress stimulation, while *Bex1* gene-deleted mice are protected from heart failure promoting insults. BEX1 directly binds selective mRNAs as part of a large riboprotein complex that stabilizes expression of disease-associated proinflammatory genes. Thus, BEX1 is an inducible and unique RNA complex-dependent disease-promoting gene in the heart.

## Results

### BEX1 is induced in heart failure and is deleterious

**Fig. 1:**
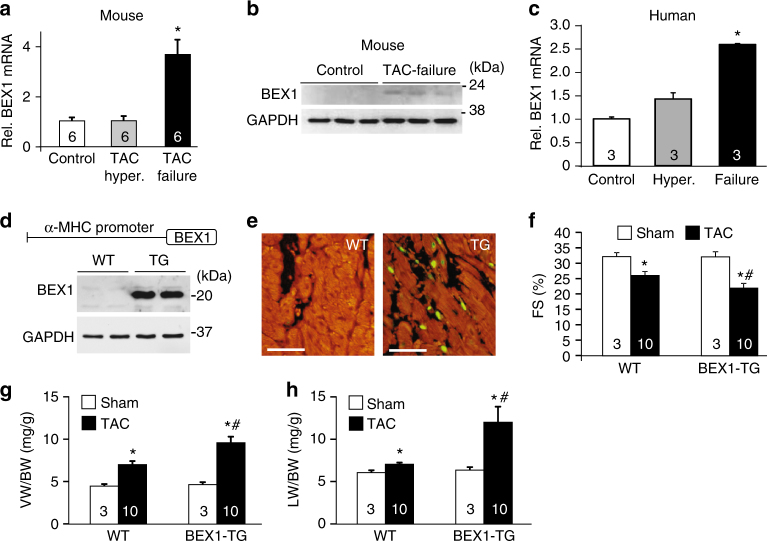
BEX1 is induced in heart failure and is deleterious. **a** RT-PCR for BEX1 mRNA levels in hearts from mice subjected to sham surgery (Control), 2 weeks pressure-overload (TAC Hyper.) or 8 weeks pressure-overload (TAC, Failure). **b** Western blot for BEX1 protein expression in cardiac extracts from mice subjected to sham surgery (Control) or 8 weeks pressure-overload (TAC Failure). GAPDH was used as loading control. Molecular weight scale is shown on the right in kiloDaltons. **c** RT-PCR for BEX1 mRNA levels in human heart samples from controls, hypertrophic subjects (Hyper.) or heart failure subjects (Failure). **d** Generation of BEX1 transgenic (TG) mice and schematic of BEX1-TG construct (upper) and western blot analysis of BEX1 expression in hearts from wild-type (WT) controls or BEX1-TG mice (lower). Molecular weight scale is shown on the right in kiloDaltons. **e** Immunofluorescence staining for BEX1 (green) and α-actinin (red) in heart histological sections from WT and BEX1-TG mice. Original magnification 200×. Scale bars are 25 µm. **f** Echocardiographic analysis of fractional shortening (FS%) in WT control and BEX1-TG mice subjected to sham or 10 weeks of TAC. **g**, **h** Ventricular weight to body weight ratio (VW/BW) and lung weight to body weight ratio (LW/BW) from WT control and BEX1-TG mice subjected to sham or 10 weeks of TAC. *P < 0.05 vs. sham; #*P* < 0.05 vs. WT TAC. *P* values are one-way ANOVA with Bonferroni correction. Number of mice analyzed is shown in the bars of each graph. All error bars represent s.e.m.

BEX1 was identified in a microarray screen for genes upregulated in the failing mouse heart^30^. We confirmed an increase in BEX1 mRNA levels in failing mouse hearts following 8 weeks of pressure overload caused by transverse aortic constriction (TAC), but not during the hypertrophic phase after 1 week of stimulation (Fig. 1a, b). Analysis of human heart samples revealed a trend towards increased BEX1 mRNA levels in hypertrophic hearts and a significant increase during failure (Fig. 1c). To investigate how this increase in BEX1 expression might impact the heart, we generated transgenic (TG) mice to express BEX1 cDNA under the control of cardiac-specific α-myosin heavy chain (α-MHC) promoter (Fig. 1d). Hearts from TG mice showed increased BEX1 expression as evidenced by Western blot analysis at 2 months of age (Fig. 1d). Immunohistochemistry from TG hearts revealed BEX1 protein mostly within the nucleus of cardiomyocytes in the uninjured heart, while hearts from wild-type (WT) non-TG mice showed almost no expression (Fig. 1e). Since BEX1-TG mice showed no overt cardiac phenotype at baseline or with aging, we simulated pathologic conditions by subjecting these mice to 10 weeks of pressure overload stimulation by TAC. Echocardiographic assessment of fractional shortening showed that BEX1-TG mice developed a more severe functional deficit compared with WT control mice (Fig. 1f), as well as greater cardiac hypertrophy and more pronounced pulmonary oedema (Fig. 1g,h), suggesting more prominent heart failure due to augmented BEX1 expression in the heart with pressure overload stimulation.

### *Bex1* deletion protects the heart from failure

**Fig. 2:**
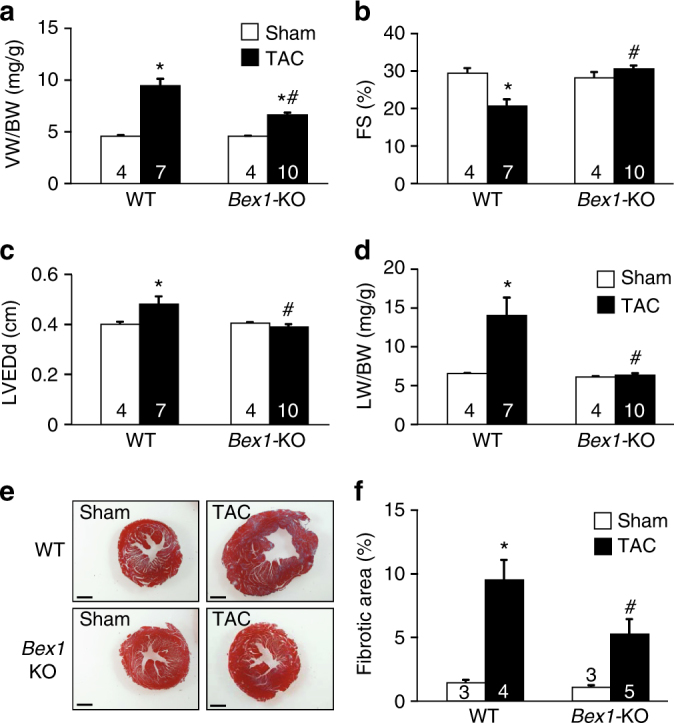
*Bex1* gene deletion protects from pressure-overload-induced heart failure. **a** VW/BW ratio from WT control and *Bex1*-KO mice subjected to sham or 5 weeks of TAC. **b** Echocardiographic analysis of fractional shortening (FS%) in WT control and *Bex1*-KO mice subjected to sham or 5 weeks of TAC. **c** Echocardiographic analysis of left ventricular end-diastolic diameter (LVEDd) in WT control and *Bex1*-KO mice subjected to sham or 5 weeks of TAC. **d** LW/BW ratio from WT control and *Bex1*-KO mice subjected to sham or 5 weeks of TAC. **e** Representative Masson’s trichrome-stained cardiac histological sections for fibrosis (blue) in mice of the indicated genotype and treatment. Original magnification is 4×. Scale bars are 0.5 mm. **f** Quantification of fibrosis from Masson’s trichrome-stained sections from WT and *Bex1*-KO hearts with sham or TAC surgery using ImageJ software. *P < 0.05 vs. sham; #*P* < 0.05 vs. WT TAC. *P* values are one-way ANOVA with Bonferroni correction. Number of mice analyzed is shown in the bars of each graph. All error bars represent s.e.m.

The data presented above suggested a deleterious role for BEX1 in the heart. To ascertain the requirement of BEX1 as an underlying effector of heart failure, we analyzed mice deleted for the *Bex1* gene (*Bex1*-KO)^25^. Similar to BEX1-overexpressing TG mice, *Bex1*-KO mice did not display a cardiac structural or functional deficit at baseline or with aging. *Bex1*-KO and control mice were thus subjected to TAC surgery, resulting in a phenotype of reduced cardiac hypertrophy after 5 weeks of pressure overload compared with WT controls (Fig. 2a). Echocardiographic assessment of ventricular performance showed that hearts from *Bex1*-KO mice had preserved cardiac function with less left ventricular chamber dilation compared with WT controls (Fig. 2b, c). *Bex1*-KO mice also were protected from developing pulmonary oedema with TAC, and they showed reduced cardiac fibrosis compared with WT controls (Fig. 2d–f). We also subjected *Bex1*-KO mice and controls to isoproterenol continuous infusion for 4 weeks as a neuroendocrine model of cardiac injury. Similar to what we observed with TAC stimulation, mice lacking the *Bex1* gene showed reduced levels of cardiac hypertrophy, no signs of lung congestion and less cardiac fibrosis (Supplementary Fig. 1). Thus, inhibition of BEX1 protein expression protected the heart from maladaptive remodeling and heart failure following pathologic insults.

### Discovery of the BEX1 interactome

**Fig. 3:**
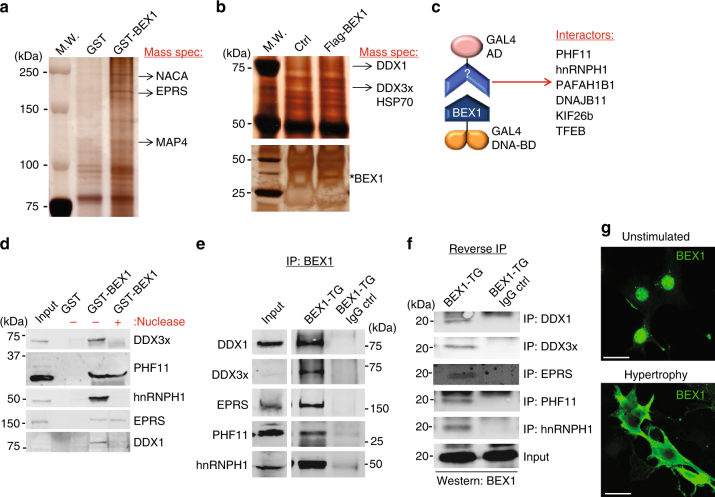
Annotating the BEX1 protein interactome. **a** Silver-stained polyacrylamide gel following GST-only or GST-BEX1 pull-down from cardiac protein extracts. Arrows indicate the binding proteins identified by mass-spectrometry. **b** Silver-stained polyacrylamide gel following control or Flag-BEX1 immunoprecipitation from neonatal rat cardiomyocytes protein extracts. Arrows indicate the binding proteins identified by mass-spectrometry. **c** Schematic of the employed yeast two-hybrid screening strategy. The arrow shows the list of binding proteins identified. **d** Western blot following GST-BEX1 pull-down of the indicated proteins from cardiac extracts of WT hearts with or without addition of micrococcal nuclease to degrade RNA. **e** Western blot for the indicated 5 proteins following immunopreciptation of BEX1 protein from BEX1 transgenic hearts. Input is shown on the left and anti-BEX1 or IgG was used for the immunoprecipitations. **f** Western blot for BEX1 protein from a BEX1 transgenic heart after immunoprecipitation of the 5 shown endogenous proteins. The input is shown on the bottom and the IgG control is shown in the right lanes. **g** Immunofluorescence for BEX1 (green) in neonatal rat cardiomyocytes in unstimulated (serum-free media) or hypertrophic conditions (media containing 2% serum). Original magnification is 100×. Scale bar is 20 µm. Abbreviations: DDX3x = DEAD box helicase 3 X-linked; DDX1 = DEAD box helicase 1; hnRNPH1 = heterogeneous nuclear ribonucleoprotein H1; PHF11 = PHD-containing factor 11; HSP70 = heat shock protein 70; TFEB = transcription factor EB; NACA = Nascent Polypeptide-Associated Complex Alpha Subunit; EPRS = Glutamyl-Prolyl-tRNA Synthetase; DNAJB11 = DnaJ Heat Shock Protein Family Member B11; PAFAH1B1 = Platelet-Activating Factor Acetylhydrolase 1b Regulatory Subunit 1; KIF26b = Kinesin Family Member 26b; MAP4 = Microtubule-Associated Protein 4. All the gels show molecular weight markers in kiloDaltons

BEX1 is a poorly characterized protein with no known functional domains or direct molecular effectors. To determine how BEX1 might function within the cell we performed three independent unbiased interaction screens. The first screen utilized a glutathione-S-transferase (GST) chromatography approach, where heart protein extracts were incubated with GST-BEX1 or GST only as a control. After elution, BEX1-specific molecular partners were visualized on silver-stained polyacrylamide gels and identified by mass spectrometry, which revealed the proteins Nascent Polypeptide-Associated Complex Alpha Subunit (NACA), Glutamyl-Prolyl-tRNA Synthetase (EPRS) and Microtubule-Associated Protein 4 (MAP4), factors involved in RNA translation or nuclear to cytoplasmic shuttling (Fig. 3a, and Supplementary Table 1). The second proteomic-based screen was performed with neonatal rat cardiomyocyte protein extracts that were infected with an adenovirus expressing a Flag-tagged version of BEX1 (Flag-BEX1) to achieve overexpression for more sensitive identification of interacting proteins. Here complexes were immunoprecipitated using antibodies against the Flag epitope, and then identified by mass-spectrometry after PAGE separation (see Methods). As a negative control, neonatal rat cardiomyocytes were infected with Adβgal and were subjected to the same immunoprecipitation protocol with Flag antibody for putative BEX1-interacting proteins. The AdBex1 infected samples specifically identified predominantly RNA-binding and regulatory factors, such as the RNA helicases DEAD-box helicase 1 (DDX1) and the DEAD box helicase 3 X-linked (DDX3x), as well as heat shock protein 70 (HSP70) (Fig. 3b, and Supplementary Table 1). Finally, we also performed a yeast two-hybrid screen with BEX1 fused to GAL4 DNA-binding domain in conjunction with a cDNA library fused to the GAL4 activation domain, which identified additional RNA interacting and regulatory factors, as well as transcriptional effectors and intracellular transport proteins (Fig. 3c). Taken together, these three independent approaches revealed that BEX1 interacts with proteins implicated in mRNA processing, translation and in microtubule-based transport inside the cell (Table 1). We confirmed these interactions in separate GST-BEX1 pull-down assays, which showed that BEX1 interacts with the transcriptional activator PHD-containing factor 11 (PHF11), the splicing factor heterogeneous nuclear ribonucleoprotein H1 (hnRNPH1), DDX3x and DDX1, as well as EPRS in the heart (Fig. 3d). Interestingly, micrococcal nuclease treatment of the cardiac protein extract prior to pull-down assay abrogated the ability of BEX1 to interact with the mRNA-binding proteins DDX1, DDX3x and hnRNPH1, suggesting the RNA-dependency for these specific molecular interactions (Fig. 3d). The lack of DDX1 signal in the absence of enrichment (input lane) is likely due to a technical problem related to the poor quality of the DDX1 antibody coupled with its rather low levels of expression in the heart.

**Table 1:** Summary of BEX1-interacting proteins assembled from 3 different screens that fit into a classification of RNA interacting, transport and maturation

Interactor	Function	Localization
DDX3x	mRNA processing	Nuclear/cytoplasmic
DDX1	mRNA processing	Nuclear/cytoplasmic
hnRNPH1	mRNA maturation	Nuclear
PHF11	mRNA synthesis	Nuclear
TFEB	mRNA synthesis	Nuclear
NACA	mRNA translation	Cytoplasmic
EPRS	mRNA translation	Cytoplasmic
DNAJB11	mRNA translation	Cytoplasmic
PAFAH1B1	Transport	Nuclear/cytoplasmic
KIF26b	Transport	Cytoplasmic
MAP4	Transport	Nuclear/cytoplasmic
HSP70	Chaperone function	Nuclear/cytoplasmic

*DDX3x* DEAD box helicase 3 X-linked, *DDX1* DEAD box helicase 1, *hnRNPH1* heterogeneous nuclear ribonucleoprotein H1, *PHF11* PHD-containing factor 11, *HSP70* heat shock protein 70, *TFEB* transcription factor EB, *NACA* nascent polypeptide-associated complex alpha subunit, *EPRS* Glutamyl-Prolyl-tRNA synthetase, *DNAJB11* DnaJ heat shock protein family member B11, *PAFAH1B1* platelet-activating factor acetylhydrolase 1b regulatory subunit 1, *KIF26b* kinesin family member 26b, *MAP4* microtubule-associated protein 4

The identified interactions were further verified in vivo in the hearts of BEX1 transgenic mice without employing a tagged version of BEX1. Here wild-type BEX1 was immunoprecipitated and western blotting was performed for DDX1, DDX3x, EPRS, PHF11 and hnRNPH1, vs. an IgG control. The data again showed an interaction between BEX1 and each of the identified endogenous proteins in the heart (Fig. 3e). These same interactions were separately probed in a reverse immunoprecipitation experiment in which specific antibodies were separately used to pull-down DDX1, DDX3x, EPRS, PHF11 and hnRNPH1 followed by western blotting for BEX1, which again confirmed each interaction (Fig. 3f).

Finally, BEX1 subcellular localization was assessed because many of the newly discovered BEX1 binding partners are known to differentially localize between the nucleus, cytoplasm or shuttle between these two compartments in affecting mRNA expressivity. In cardiomyocytes BEX1 localized predominantly in the nucleus under resting conditions, but shuttled to the cytoplasm in response to hypertrophic agonist stimulation (Fig. 3g). Thus, BEX1 appears to be part of a gene-expression regulating complex that underlies mRNA processing and expressivity in transit between the nucleus and cytoplasm (see below).

### BEX1 regulates proinflammatory genes

**Fig. 4:**
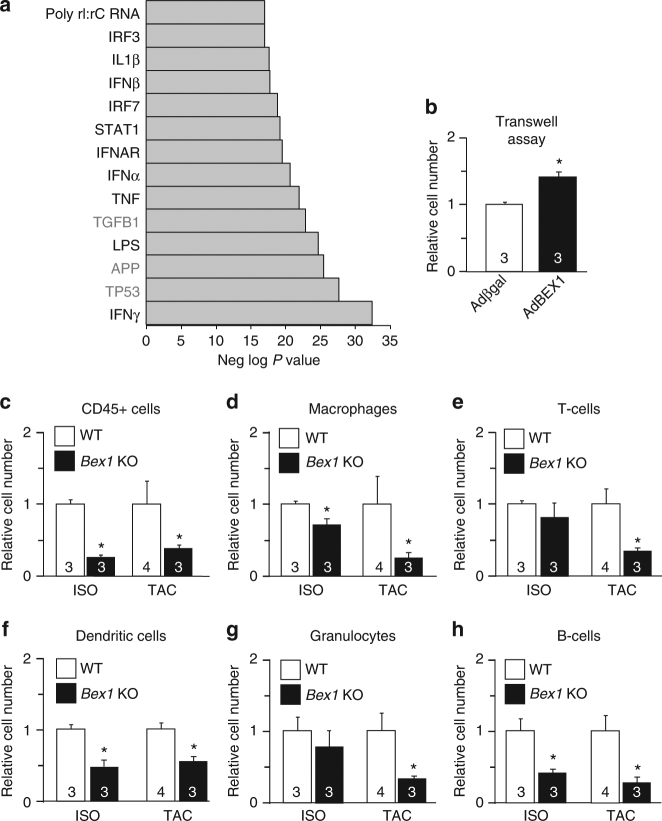
BEX1 regulates proinflammatory mRNAs. **a** Ingenuity pathway analysis of RNA-seq from neonatal rat cardiomyocytes overexpressing BEX1 compared to β-galactosidase-overexpressing control (adenoviral mediated). Abbreviations: IFNγ (interferon-γ); TP53 (tumour suppressor p53); APP (amyloid precursor protein); LPS (lipopolysaccharide); TGFβ1 (transforming growth factor-β1); TNF (tumour necrosis factor); IFNα (interferon-α); IFNAR (interferon α/β receptor); STAT1 (signal transducer and activator of transcription 1); IRF7 (interferon response factor 7); IFNβ (interferon β); IL1β (interleukin-1β); IRF3 (interferon response factor 3); poly rI:rC RNA (polyriboinosinic acid and polyribocytidylic acid RNA). **b** Trans-well migration assay of immune cells attracted by neonatal rat cardiomyocytes overexpressing BEX1 compared vs. β-galactosidase-overexpressing control. **c**–**h** Quantification of the type of immune cell shown from the mouse heart following isoproterenol infusion (ISO) or pressure overload stimulation (TAC) by FACS using antibodies against CD45 (CD45 + cells), CD11b/MAC1 (Macrophages), CD3e (T-cells), CD11c (Dendritic cells), Ly6G (Granulocytes) and CD45R/B220 (B-cells). *P < 0.05 vs. WT. *P* value is unpaired 2-tailed *t* test. Number of mouse hearts analyzed is shown in the bars of each graph. All error bars represent s.e.m.

Given the interaction data discussed above, we next investigated if BEX1 might be modulating mRNA levels in the heart by RNAseq analysis. Neonatal rat cardiomyocytes were infected with a BEX1-expressing recombinant adenovirus and RNA was collected, processed and sequenced, which showed a unique profile of proinflammatory and interferon-regulated genes (Fig. 4a, black text). To validate and extend these observations, we selected a number of candidate genes from our RNAseq data to assess in pressure overloaded hearts from WT vs. *Bex1*-KO mice. We observed that in the absence of the *Bex1* gene there was less induction of proinflammatory and interferon-stimulated mRNAs, such as interferon-stimulated gene 15, (Isg15), C-X-C motif chemokine 10 (Cxcl10) and tumour necrosis factor-α (TNFα) in the injured myocardium vs. WT mice (Supplementary Fig. 2a–c). In addition, we isolated mouse embryonic fibroblasts (MEFs) from *Bex1*-KO and control WT mice and stimulated these cells with lipopolysaccharide (LPS), a potent inducer of innate immunity and interferon-dependent inflammation. Remarkably, we observed by RT-PCR analysis that MEFs deleted for *Bex1* had significantly reduced levels of Isg15 and Cxcl10 after stimulation with LPS in culture (Supplementary Fig. 2d, e).

Given the ability of BEX1 to seemingly enhance proinflammatory cytokine gene expression, we assessed the ability of cardiomyocytes overexpressing BEX1 to chemo-attract immune cells. In agreement with our RNA profiling findings, we observed that increasing BEX1 expression in cardiomyocytes favoured the migration of immune cells in a trans-well migration assay (Fig. 4b). We also examined if inhibition of BEX1 might prevent or reduce a cellular proinflammatory signature in the heart after injury. For this analysis we quantified various immune cells infiltrating the heart by FACS analysis after pressure overload stimulation or isoproterenol infusion. The data show that in hearts from *Bex1*-KO mice there were significantly reduced levels of resident CD45 cells, macrophages, T-cells, dendritic cells, granulocytes and B-cells with either stimulation protocol compared with WT (Fig. 4c–h). Thus, BEX1 regulates a proinflammatory gene-expression program in the heart that can coordinate the abundance of immune cells during an injury response.

### BEX1 controls A + U-rich element-containing mRNAs

**Fig. 5:**
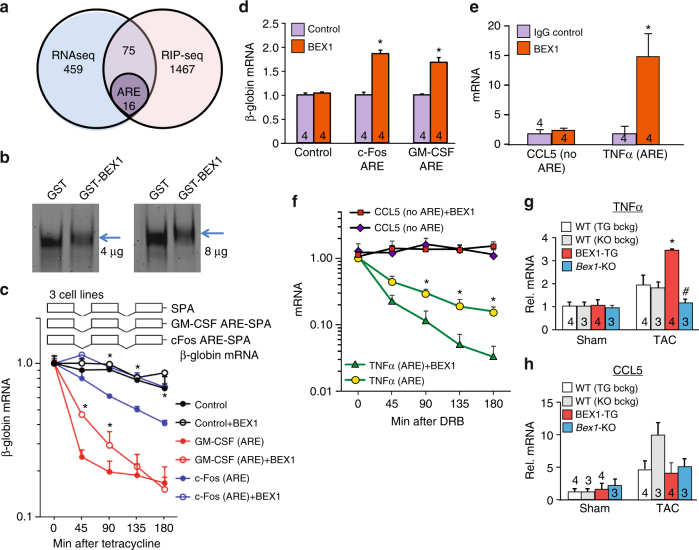
BEX1 directly controls A + U-rich element containing mRNAs. **a** Comparison of RNAseq and RIP-seq list showing 91 overlapping mRNAs due to BEX1 overexpression or BEX1-dependent pull-down, respectively, 17.58% of which contains AU-rich elements (ARE). **b** RNA EMSA showing an ARE-ribonucleoprotein cardiac complex shift from heart protein extract at 4 and 8 μg, which is shifted even higher by addition or recombinant BEX1-GST (blue arrows). GST was used as negative control. **c** qPCR for β-globin mRNA at 0, 45, 90, 135 and 180 min post-tetracycline addition in stable cell lines (control, GM-CSF ARE or *c-fos* ARE) transfected with BEX1 or GFP control plasmid. A synthetic poly A (SPA) is present in all 3 constructs. Results reflect 3 separate experiments. **d** Level of β-globin mRNA following RNA immunoprecipitation using Flag antibodies from Flag-BEX1 or GFP expressing stable cell lines (control, GM-CSF ARE and c-fos ARE) by qPCR. The results are from 3 separate experiments. **e** qPCR analysis for TNFα and CCL5 mRNA immunoprecipitated with BEX1 antibody from BEX1 transgenic hearts. Number of experiments conducted is shown in the graph for each condition. *P < 0.05 vs. IgG control. **f** qPCR for TNFα and CCL5 gene products at 0, 45, 90, 135, and 180 min after DRB-mediated inhibition of transcription in neonatal rat cardiomyocytes infected with BEX1 or β-galactosidase control adenoviruses. The average from 3 independent experiments is shown. **g**, **h** qPCR analysis of TNFα mRNA (**g**) and CCL5 mRNA **h** from hearts of BEX1-TG, WT (TG bckg), *Bex1*-KO and WT controls (KO bckg) subjected to TAC or sham surgeries. Bckg = strain background. Number of hearts analyzed is shown in the graph for each condition. *P < 0.05 vs. WT (TG bckg) TAC mRNA. #*P* < 0.05 vs. WT (KO bckg) TAC mRNA. For all other panels *P < 0.05 vs. ctrl. All error bars represent s.e.m. All *P* values are unpaired 2-tailed *t* test for this figure

To identify mRNAs that are directly regulated by BEX1, RNA immunoprecipitation (RIP) assays were performed followed by sequencing (RIP-seq). Neonatal rat cardiomyocytes were first infected with either a control adenovirus or one encoding Flag-BEX1, and then Flag antibody was used to pull-down BEX1 to identify associated mRNAs by sequencing (Fig. 5a and Supplementary Dataset). We observed that approximately 20% of the mRNAs that were associated with the BEX1 complex (overlap) contained A + U-rich elements (ARE) within their 3’ untranslated region (UTR) (Fig. 5a). Within the overlapping genes, 43% of the mRNAs were upregulated upon BEX1 overexpression, while 57% were downregulated. Indeed, RNA-binding proteins can target ARE elements in mRNAs and exert stabilizing or destabilizing effects. Critical proinflammatory mRNAs, such as interferon family member genes, TNFα or interleukin-1β (Fig. 4a; major pathways affected by BEX1) contain AREs that regulate their stability as means of post-transcriptional gene expression modulation.

To more directly examine if BEX1 binds ARE sequences in the heart, we performed a series of RNA-electrophoretic mobility shift assay (EMSA) experiments. We observed that BEX1 is able to shift the predominant ARE-ribonucleoprotein complex that forms with mouse heart protein extracts (Fig. 5b). However, recombinant BEX1-GST did not act as a site -specific RNA-binding factor to the ARE in these EMSA experiments, which is consistent with a lack of a known RNA-binding motif in BEX1 (data not shown). To assess if BEX1 is a novel regulator of mRNA decay through AREs, we adopted a system where mouse fibroblasts stably express tetracycline-regulated plasmids encoding either a control β-globin mRNA with no added instability elements or β-globin mRNA with the GM-CSF or *c-fos* AREs in their 3’-UTRs, as depicted in Fig. 5c (upper panel; these constructs contain a synthetic poly A, SPA)^31^. In this system, β-globin transcription can be turned off by addition of tetracycline to the culture media, allowing for mRNA decay over time. The use of the two separate classes of AREs, one from the GM-CSF and one from *c-fos* mRNA, allows for a more thorough examination of how BEX1 might affect message stability through these 2 classes of ARE elements. We observed that BEX1 stabilized the chimeric β-globin for both classes of AREs, but had no effect on a nonARE-containing control message (Fig. 5c). To assess if BEX1 is complexed with these ARE-containing mRNAs, we transfected Flag-BEX1 and used antibodies against the Flag epitope to immunoprecipitate BEX1 from the stable cell lines expressing β-globin control, β-globin GM-CSF ARE or β-globin *c-fos* ARE. qPCR analysis showed that BEX1 complexed with β-globin message only when either class of ARE were present (Fig. 5d).

BEX1 was also determined to associate with ARE-containing mRNAs in the heart. Specifically, hearts from BEX1 transgenic mice were collected followed by RNA immunoprecipitation with a BEX1 antibody, followed by RNA extraction and qPCR analysis. We observed association of BEX1 with mRNAs for TNFα, a classical proinflammatory ARE-messenger, while mRNAs missing the AU-rich element, such as C-C motif chemokine ligand 5 (CCL5), were not associated with BEX1 (Fig. 5e). These increased levels of mRNAs were likely due to a mechanism involving RNA stability. For example, mRNA levels were analyzed in neonatal rat cardiomyocytes following treatment with the transcription inhibitor DRB (5,6-dichloro-1-β-D-ribofuranosylbenzimidazole), which showed that BEX1 directly stabilized the ARE-containing mRNA message for TNFα, but not CCL5 (Fig. 5f). Delay in poly A shortening can also contribute to BEX1-mediated stabilizing of ARE-mRNAs, as TNFα-ARE deadenylation experiments revealed some protection from poly A degradation by BEX1 (data not shown). Finally, we extended these regulatory relationships to an in vivo context in the hearts of BEX1-TG mice vs. *Bex1*-KO mice, at baseline and after TAC stimulation. While the nonARE-containing CCL5 transcript was not affected, we observed that the ARE-containing TNFα transcript was significantly induced in the hearts of BEX1-TG mice after TAC compared with WT controls subjected to TAC, while it was significantly inhibited in the hearts of *Bex1*-KO mice compared with their strain-matched controls (Fig. 5g, h). Taken together, these results suggest that BEX1 is a direct regulator of ARE-containing mRNA stability, which likely underlies its role in augmenting inflammatory signalling in the injured heart.

To further investigate the mechanism whereby BEX1 can stabilize ARE-containing mRNAs, the potential dependence of the known BEX1 RNA-binding proteins presented in Fig. 3 were investigated for BEX1-dependent effects on TNFα mRNA levels. Neonatal rat cardiomyocytes were infected with AdBEX1 and selected siRNAs against either PHF11, hnRNPH1, DDX1, DDX3 or EPRS (Supplementary Fig. 3). The data show that the BEX1-dependent increase in TNFα mRNA was lost with siRNA against DDX1, suggesting that this RNA helicase was a necessary component of the BEX1 interactome in mediating the effects of BEX1 on mRNA stability of ARE-containing messages.

## Discussion

The immune system evolved to provide a global defense against pathogens, as well as to sense tissue injury. Although the activation of immune cells can be beneficial by promoting healing and the efficient destruction of invading pathogens, the inherent cost can be an augmentation in general inflammatory damage to tissues that are already damaged or part of some sort of acquired disease sequelae^32^. Recent studies suggest that the heart possesses an innate immunity response that can be initiated after myocardial injury by a family of pattern recognition receptors (PPRs) including CD14, a pattern recognition receptor for LPS, as well as Toll-like receptors (TLRs)^3, 33, 34^. The result of PPR activation is the trigger of a sterile inflammatory response mediated by the release of cytokines and interferon. While this system undoubtedly evolved as a short-term adaptive response to tissue injury, the beneficial effect of this phylogenetically ancient system is likely lost when activation becomes excessive and sustained^34^. Recent observations of dysregulated activation of this type of response in heart failure patients raise the interesting possibility of targeting this pathway as a novel therapeutic approach^35, 36^.

Here we identified BEX1 as a novel intracellular regulator of the inflammatory response, which is detrimental to the heart and responsible for disease progression during chronic pathologic insults. Our results showed that BEX1 expression is induced in the injured heart, and that deleting the *Bex1* gene reduced or prevented cardiomyopathy with chronic stress stimulation. Indeed, forced expression of BEX1 in cardiomyocytes worsened pressure-overload-induced cardiac dysfunction and remodeling. To our knowledge, this is the first report demonstrating a causative role of BEX1 in cardiac disease as a proinflammatory factor. Interestingly, the identification of BEX1 as an inflammatory effector is consistent with previously reported roles for BEX1 in axonal and skeletal muscle regeneration, as well as in tumourigenesis^24, 25, 27^. For example, suppression of inflammation after nerve injury compromises axon regeneration; and activation of the immune response is necessary for proper skeletal muscle regeneration post-injury^37–40^. Also, inflammation is now considered a dominant feature and a hallmark of cancer, and oncogenes and tumour suppressors are known regulators of inflammatory pathways^41–43^. It is also intriguing to note that Vilar and colleagues observed that BEX1 interferes with nerve growth factor signalling in neuronal cells and indirectly affects the activity of NF-κB, a transcription factor that regulates the expression of proinflammatory genes^26^.

Our 3 levels of unbiased screens for BEX1 interactors revealed that this protein is part of an RNA processing and shuttling complex with components acting at multiple steps of the mRNA generation and maturation process from transcription in the nucleus to translation at the ribosomes in the cytoplasm. Indeed, BEX1 shuttles from nucleus to cytoplasm with stress stimulation, suggesting that it can follow target mRNAs during their life and stabilize them through the process of translation. The interaction with DDX1, a factor that also shuttles with mRNA as it moves from the nucleus to the cytoplasm, was shown to be required for the effect of BEX1 on TNFα mRNA levels and stability. This result provided further mechanistic understanding of the relevance for BEX1 interacting with various other RNA-binding proteins and how this entire complex functions in regulating inducible gene expression in the heart.

Our screen revealed the association of BEX1 with proteins functioning in microtubule-mediated transport. Interestingly, a recent report has identified BEX4, another *Bex* gene family member, as a microtubule-associated protein regulating tubulin dynamics^44^. It is known that ribonucleoprotein particles travel inside the cell along microtubule-dependent routes^45, 46^, and BEX1 could provide specificity for a subset AU-rich element (ARE) containing mRNAs that underlie the inflammatory response^18^. However, we also observed that a subset of BEX1-bound mRNAs were significantly downregulated with BEX1 overexpression, suggesting additional layers of complexity to the biology of how BEX1 functions in the heart as an mRNA effector.

Interestingly, essentially all the binding partners of BEX1 have been previously implicated in immune system regulation. A role for PHF11 in allergic reactions has been recently proposed, and this factor was shown to cooperate with NF-κB in promoting interleukin 2 and interferon gamma (IFNγ) expression in T-lymphocytes^47^. The RNA helicase DDX1 was shown to act as a viral sensor activating type I interferon responses^48^. DDX3x is perhaps the best-characterized factor of all the BEX1 interactors that we identified, which is involved in RNA metabolism in both the nucleus and cytoplasm, and it is a known host target for viral replication^49, 50^. DDX3x is also a critical component of the interferon-dependent proinflammatory response by favouring IFNβ expression^51, 52^. The tRNA synthetase EPRS was also implicated in interferon signalling, such as being a part of the IFNγ-activated inhibitor of translation (GAIT) system, which is a complex that directs transcript-selective translational control in myeloid cells in response to IFNγ^53, 54^. To our knowledge, our observations are the first to link these various immune modulators together as components of the same molecular RNA-regulating complex, suggesting a direct molecular function for BEX1 and perhaps the entire family of BEX genes.

## Methods

### Mice

A tetracycline-responsive binary α-myosin heavy chain (α-MHC) transgene system was used to temporally regulate expression of BEX1 in cardiomyocytes^55^, although because constitutive expression of BEX1 was of no demonstrable effect, the inducibility feature was never employed or discussed. These mice were generated in FVB/N background by standard pronuclear injection of the DNA construct into newly fertilized mouse embryos. The generation of *Bex1* gene-deleted mice was realized by Dr. Margolis at the University of Maryland and previously described^25^. Eight to ten-week old male and female mice were used in every experiment with the exception of pressure overload surgeries on *Bex1* gene-deleted mice where males only were used to reduce variability. All experiments involving animals were approved by the Institutional Animal Care and Use Committee at Cincinnati Children’s Hospital Medical Center. No human subjects were used. Mice were either sacrificed by CO_2_ asphyxiation or by excision of the heart under deep isoflurane sedation for final analysis of tissue.

### Human samples

Control heart samples were obtained from healthy donors while hypertrophied hearts were taken from patients who were initially considered to be donors, with no history or findings of congestive heart failure, but who by post-explant examination revealed cardiac hypertrophy^56^. Heart failure samples were obtained from hearts explanted from patients with heart failure before cardiac transplantation^56^. The use of these previously obtained human heart samples was approved by the Institutional Review Board at Cincinnati Children’s Hospital Medical Center and consent was obtained from all subjects.

### Mouse surgery and physiology assessments

All mice were anaesthetized with 2% Isofluorane by inhalation. Echocardiography was performed in M-mode using a Hewlett Packard SONOS 5500 instrument equipped with a 15 MHz transducer as described previously^57^. Cardiac hypertrophy was induced by transverse aortic constriction (TAC) to produce pressure overload as previously described^58^. Doppler echocardiography was performed on all mice subjected to TAC to ensure equal pressure gradients across the aortic constriction between the groups. Infusion of isoproterenol (60 mg/kg/day) was performed with implantation of Alzet minipumps for 4 weeks (Durect Inc).

### Cell and tissue analysis and immunofluorescence

Neonatal rat ventricular cardiomyocytes were isolated as previously published^59^. Mouse embryonic fibroblasts (MEFs) were isolated from day 15.5 to 16.5 embryos from WT and *Bex1*-KO mice. Masson’s trichrome staining for fibrosis (blue) was performed from heart histological sections generated with paraffin-embedding. Immunohistochemistry was performed on cryosections from OCT (Tissue-Tek) embedded hearts. Cardiomyocytes were stained using antibodies recognizing α-actinin (Sigma-Aldrich; A2172; mouse monoclonal; diluted 1:300). BEX1 was visualized using an antibody from Abcam (ab68936; rabbit polyclonal; diluted 1:200). Antibodies against the Flag epitope (Sigma-Aldrich; F1804; mouse monoclonal; diluted 1:200) were used in immunofluorescence experiments to visualize Flag-BEX1 in paraformaldehyde-fixed neonatal rat cardiomyocytes.

### Western blotting and mRNA expression analysis

Western blotting was performed from mouse hearts homogenized in RIPA buffer (50 mM Tris-HCl, 1 mM EDTA, 1 mM EGTA, 150 mM NaCl and 1% NP-40) containing protease inhibitor cocktail (Sigma-Aldrich P8340) with a Dounce homogenizer. Antibodies used were BEX1 (Abcam; ab68936; mouse monoclonal; diluted 1:500) and GAPDH (Fitzgerald Industries; R-G109a; mouse monoclonal; diluted 1:20000). RNA was extracted from ventricles or neonatal rat cardiomyocytes using the RNeasy kit according to manufacturer’s instructions (Qiagen). Uncropped western gel images are shown in Supplementary Fig 4.

Neonatal rat cardiomyocyte samples overexpressing BEX1 or β-galactosidase (control) by adenoviral infection were submitted for RNAseq (University of Cincinnati sequencing and genome analysis core laboratory). For RNAseq data analysis, *P* values were calculated using a negative binomial statistical model as implemented in DESeq [Bioconductor version: Release (2.14)], meanwhile false discover rates were used to obtain the adjusted *P*-values. Ingenuity Pathway Analysis software was utilized to identify the significantly affected pathways. The RNAseq data were deposited in the GEO database, https://www.ncbi.nlm.nih.gov/geo/ (accession #GSE95523). siRNA treatment was performed on neonatal cardiomyocytes using lipofectamine-mediated transfection. Targeted mRNAs included PHF11 (sc-152207), DDX1 (sc-60518), EPRS (sc-76255), DDX3x (sc-77109) and hnRNPH1 (sc-35580). For qPCR analysis, reverse transcription was performed using the High Capacity cDNA Reverse Transcription kit (Applied Biosystems). Selected gene expression differences were analyzed by real-time qPCR using SYBR green (Applied Biosystems). Quantified mRNA expression was normalized to Rpl7 (Ribosomal Protein L7), Actb (Beta-Actin) or Sdha (Succinate Dehydrogenase Complex Flavoprotein Subunit A), and expressed relative to controls. Primers used were: hBex1 5′-GGGAGAAGGAGGAGACTACAA-3′ and 5′-TCCATGCTGAGACTGTTTACTG-3′; hHbb 5′-TGGATGAAGTTGGTGGTGAG-3′ and 5′-CCTTAGGGTTGCCCATAACA-3′; hSdha 5′-CATGCAGGCCTGGAGATAAA-3′ and 5′-GTCGCAGTTCCGATGTTCTTA-3′; mActb 5′-TGTGATGGTGGGAATGGGTCAGAA-3′ and 5′-TGTGGTGCCAGATCTTCTCCATGT-3′; mTnfa 5′-TCACTGGAGCCTCGAATGTC-3′ and 5′-GTGAGGAAGGCTGTGCATTG-3′; mCcl5 5′-TGCAGAGGACTCTGAGACAGC-3′ and 5′-GAGTGGTGTCCGAGCCATA-3′; mIsg15 5′-GGTGTCCGTGACTAACTCCAT-3′ and 5′-CTGTACCACTAGCATCACTGTG-3′; mCxcl10 5′-GCTGCCGTCATTTTCTGC-3′ and 5′-TCTCACTGGCCCGTCATC-3′; and mRpl7 5′-TGGAACCATGGAGGCTGT-3′ and 5′-CACAGCGGGAACCTTTTTC-3′.

### Protein interaction screening

Co-immunoprecipitation was performed in rat neonatal cardiomyocytes infected with adenoviral vectors encoding β-galactosidase (control) or Flag-BEX1. Antibodies against the Flag epitope (Sigma-Aldrich; F1804) were used to precipitate BEX1 interactors. Co-immunoprecipitation from mouse hearts was performed using antibodies against BEX1 (kindly provided by Dr. Frank Margolis; rabbit polyclonal; used 1:2000 for visualization by Western Blot), DDX1 (Proteintech 11357-1-AP; rabbit polyclonal; diluted 1:1000 for visualization by Western Blot), DDX3x (Bethyl Laboratories A300-474A; rabbit polyclonal; diluted 1:1000 for visualization by Western Blot), PHF11 (Millipore ABE396; rabbit polyclonal; diluted 1:1000 for visualization by Western Blot), EPRS (Abcam ab31531; rabbit polyclonal; diluted 1:1000 for visualization by Western Blot), hnRNPH1 (Abcam ab10374; rabbit polyclonal; diluted 1:1000 for visualization by Western Blot), or normal IgG control. Recombinant GST and GST-BEX1 proteins were produced in E. Coli and incubated with mouse ventricular protein extracts for pull-down of molecular partners with or without Micrococcal nuclease (Sigma-Aldrich; N3755). For analysis of BEX1 protein interactions from neonatal rat cardiomyocytes, cells were infected with adenoviral vectors encoding β-galactosidase (control) or Flag-BEX1, and antibody against the Flag epitope (Sigma-Aldrich; F1804; mouse monoclonal) was used. Yeast two-hybrid screening was performed using the GAL4 system as previously published^60^. A universal mouse cDNA library was purchased from Takara Clontech (630482).

### Protein identification by mass spectrometry

Following GST pull-down or co-immunoprecipitation, the proteins were run on polyacrylamide gels and silver staining was performed. Selected bands were excised from the gel, destained and dehydrated, then subjected to a tryptic digestion as originally described by Shevchenko (1996) with modification as presented by Eismann and colleagues in 2009^61, 62^. After digestions, the extracted peptides were concentrated in a Speed-Vac to a final volume of 10–15 µL. The peptides were desalted and purified utilizing the C_18_ ZipTips (Millipore, Bedford, MA, USA) and eluted in 2.5 µL of 0.1% formic acid in 60% acetonitrile, followed by peptide concentration to a final volume of 0.5–1.0 µL in the SpeedVac. One µL of matrix solution (0.1% formic acid in 60% acetonitrile + 5 mM ammonium phosphate monobasic + 10 µg/µL α-cyano-4-hydroxy-cinnamic acid) was mixed with the peptides and directly loaded onto a matrix assisted laser desorption/ionization (MALDI) target plate (Opti-TOF 384 well plates from Sciex). MALDI-MS analysis of each digestion was performed on a Sciex 4800 MALDI-TOF-TOF instrument operated in positive ion reflector mode with a laser attenuation set to 3800. Peptide spectra were averaged from 1000 laser shots, and the top 15 peptide signals from each spectrum were subsequently subjected to MS/MS fragmentation for sequence analysis. Fragmentation spectra were collected at a laser attenuation of 4200 from 2000 laser shots for each peptide***. Proteins were identified using the MASCOT search algorithm (Matrix Science) from a combination of peptide mass fingerprint (PMF) profiles and MS/MS sequencing of up to 15 peptides for each digestion. Search parameters were limited to all mammalian sequences (1,041,092 proteins for the NCBI-nr database downloaded on 1/23/2012); Trypsin digestion was performed with up to 2 missed cleavages, 125 ppm peptide mass tolerance, 0.8 Da fragment mass tolerance with carbamidomethyl cysteine as a fixed modification and oxidized methionine as a variable modification. Criteria for confident protein identification included a protein *p* value < 0.005, a minimum of 2 MS/MS spectra of sufficient quality to surpass a Mascot peptide identity score and/or a minimum protein score of 90. Identified peptide sequences are provided as Supplementary Table 1. Most protein identifications were verified with an independent biochemical assay such as endogenous immunoprecipitations or GST pull-downs followed by antibody-based recognition.

### FACS analysis and trans-well migration assay

FACS analysis was performed on cardiac interstitial cells as described before^63^. Briefly, mouse cardiac ventricles were digested in collagenase type 2 (Worthington LS004177). After the digestion, cardiomyocytes and debris were eliminated by two serial centrifugations at 10 g for 5 min at 4 °C and the non-cardiomyocyte cell fraction was collected after a final centrifugation at 500 g for 10 min at 4 °C and pellets were resuspended in 2% fetal calf serum in HBSS. Flow cytometry analysis was performed using a BD FACSCanto II running FACSDiva software (BD Biosciences). Analysis was performed using FlowJo vX (FloJo). Cardiac interstitial cells were stained with surface markers using APC conjugated antibodies against CD45 (BD Biosciences 559864); CD11C (eBioscience 17-0114-81) or biotinylated CD45R (B220); CD11b (Mac1); Ly-6G and CD3e from mouse hematopoietic lineage panel kit (eBioscience 88-7774-75) combined with APC conjugated streptavidin (eBioscience 17-4317-82). For trans-well migration analysis, neonatal rat cardiomyocytes were seeded in gelatin coated 24-well plates and infected with adenoviruses expressing β-galactosidase (control) or BEX1. Migration assays were performed in 6.5 mm Transwell plates (Sigma-Aldrich) with 8 μm pore inserts. The upper side of one insert was thinly coated for 1 h with rat tail type I collagen (Sigma-Aldrich). J774A.1 cells (1 × 10^5^ cells) were added to the upper chamber in 600 μl serum-free DMEM medium. Cells were allowed to migrate through the insert membrane for 3 h at 37 °C, 5% CO_2_. The inserts were then washed with PBS and non-migrating cells on the upper surface of the insert were removed with a cotton swab. The migrated cells on the bottom of the insert were labelled with 1 μM calcein AM for 15 min, visualized with a stereomicroscope with fluorescence capability and counted.

### Gating strategy for FACS

To determine optimal flow cytometry voltages, we used single-stain and fluorescence minus one (FMO) controls as well as unstained bone marrow and wild-type cardiac interstitial cells. For the analyses of subpopulations of cardiac interstitial cells, a series of gating strategies were employed. The total cardiac interstitial cell suspension was first gated using pulse signals detected with Forward Scatter Area (FSC-A) vs. Side Scatter Area (SSC-A) to separate debris from total cells. Next, two consecutive selection gates with Side Scatter Width (SSC-W) against height (SSC-W) and Forward scatter width (FSC-W) against height (FSC-H) were utilized to remove doublet events or aggregates from the analysis. The final single cell interstitial population was verified to represent live cells by using either 7AAD (Thermo Fisher Scientific Cat # A1310) or Calcein Blue, AM (Thermo Fisher Scientific Cat. # C1429) in separate flow cytometry runs. The final population was then analyzed by gating for antibodies conjugated to the APC fluorophore signal as described above. In addition, a back-gating analysis was performed to confirm the specificity and efficiency of the gating strategy to ensure inclusion of all of the cells of interest.

### RNA analysis and RNA immunoprecipitation

For RNA EMSA 1 pmol Cy5 labelled ARE-TNF-cy5 (Cy5-gcacttattatttattatttatttattatttatttatttgcttatgaatgtatttatttg-3′) RNA probe (Dharmacon) was incubated with GST or GST-BEX1 protein together with 4 μg or 8 μg of cytoplasmic heart extracts in binding buffer (10 mM Tris-HCL, 50 mM KCl, 2.5 mM DTT, 0.25% Tween 20, pH 7.5, plus RNase inhibitor). Reaction mixtures were incubated at room temperature for 30 min and resolved on a non-denaturating 5% TBE gel and RNA-protein complexes were visualized by a LICOR Odyssey infrared scanner. mRNA decay experiments were performed in LM(tk-tTA) murine cell lines stably expressing the human β-globin gene with a synthetic poly A (SPA) and without added instability element or with *c-fos* or GM-CSF ARE as previously described^31^. mRNA decay experiments in cardiomyocytes were performed using 5,6-dichloro-1-β-D-ribofuranosylbenzimidazole (DRB; Sigma-Aldrich) as a transcription inhibitor. For RNA immunoprecipitation of ARE-mRNAs, the cells were fixed in 0.15% formaldehyde prior to standard pull-down with anti-Flag M2 magnetic beads (M8823, Sigma) and phenol-chloroform precipitation of associated RNA. For RNA immunoprecipitation (RIP) from mouse hearts, the tissue was chopped and submerged in RNA Later (ThermoFisher; AM7020) prior to fixation in 0.15% formaldehyde, followed by standard pull-down with antibody against BEX1 (kindly provided by Dr. Frank Margolis) or normal IgG control. For RIP-sequencing, Magna RIP kit (Millipore) was used to extract RNA, and anti-Flag M2 magnetic beads (M8823, Sigma) were used to immunoprecipitate ribonucleoprotein complexes from neonatal rat cardiomyocytes overexpressing Flag-tagged BEX1 or β-galactosidase control. Eluted RNAs were detected by RNAseq analysis. ARE database search engine (http://brp.kfshrc.edu.sa/AredOrg/AREDServlet) was used to determine the percentage of ARE-containing hits within the mRNAs bound by BEX1. The entire data set of RIP-based gene sequencing is shown in the Supplementary Data set.

### Statistics

All results are presented as mean ± SEM. Statistical analysis was performed with unpaired two-tailed *t* test (for 2 groups) and 1-way ANOVA with Bonferroni correction (for groups of 3 or more). *P* values less than 0.05 were considered significant. For studies involving cardiac injury such as TAC or isoproterenol infusion, group sizes were determined based on previously observed post-operative mortality rates for this procedure. No experimental animals were excluded in any of the analyses. Animal numbers and sample sizes reflected the minimal number needed for statistical significance based on power analysis and prior experience. No data were excluded from any of the experiments, and randomization was performed for the surgical groups. However, full blinding was not performed given logistical issues with animals under post-surgical veterinary care and the various levels of barrier housing that was used.

### Data availability

The Supplemenatary Data sets contain all the raw mRNA immuopreciptiation data. The RNAseq data set was deposited in GEO under accession code GSE95523. All other raw data will be made available upon reasonable request.

## Electronic supplementary material

Supplementary Information

Description of Additional Supplementary Files

Supplementary Data 1

## References

[1] Pugh PJ, Jones RD, Jones TH, Channer KS (2002). Heart failure as an inflammatory condition: potential role for androgens as immune modulators. Eur. J. Heart Fail..

[2] Frangogiannis NG (2014). The inflammatory response in myocardial injury, repair, and remodelling. Nat. Rev. Cardiol..

[3] Mann DL (2015). Innate immunity and the failing heart: the cytokine hypothesis revisited. Circ Res.

[4] Maier HJ (2012). Cardiomyocyte-specific IkappaB kinase (IKK)/NF-kappaB activation induces reversible inflammatory cardiomyopathy and heart failure. Proc. Natl Acad. Sci. USA.

[5] Deng KQ (2016). Suppressor of IKKvarepsilon is an essential negative regulator of pathological cardiac hypertrophy. Nat. Commun..

[6] Butts B, Gary RA, Dunbar SB, Butler J (2015). The Importance of NLRP3 Inflammasome in Heart Failure. J. Card. Fai.l.

[7] Fenton KE, Parker MM (2016). Cardiac function and dysfunction in sepsis. Clin. Chest. Med..

[8] Beckmann BM, Castello A, Medenbach J (2016). The expanding universe of ribonucleoproteins: of novel RNA-binding proteins and unconventional interactions. Pflugers Arch..

[9] Glisovic T, Bachorik JL, Yong J, Dreyfuss G (2008). RNA-binding proteins and post-transcriptional gene regulation. FEBS Lett..

[10] Singh G, Pratt G, Yeo GW, Moore MJ (2015). The clothes make the mRNA: past and present trends in mRNP fashion. Annu. Rev. Biochem..

[11] Gao C (2016). RBFox1-mediated RNA splicing regulates cardiac hypertrophy and heart failure. J. Clin. Invest..

[12] Guo W (2012). RBM20, a gene for hereditary cardiomyopathy, regulates titin splicing. Nat. Med..

[13] Koshelev M, Sarma S, Price RE, Wehrens XH, Cooper TA (2010). Heart-specific overexpression of CUGBP1 reproduces functional and molecular abnormalities of myotonic dystrophy type 1. Hum. Mol. Genet..

[14] Dixon DM (2015). Loss of muscleblind-like 1 results in cardiac pathology and persistence of embryonic splice isoforms. Sci. Rep..

[15] Davis J (2015). MBNL1-mediated regulation of differentiation RNAs promotes myofibroblast transformation and the fibrotic response. Nat. Commun..

[16] Suresh Babu S, Joladarashi D, Jeyabal P, Thandavarayan RA, Krishnamurthy P (2015). RNA-stabilizing proteins as molecular targets in cardiovascular pathologies. Trends Cardiovasc. Med..

[17] Gingerich TJ, Feige JJ, LaMarre J (2004). AU-rich elements and the control of gene expression through regulated mRNA stability. Anim. Health Res. Rev..

[18] Khabar KS (2010). Post-transcriptional control during chronic inflammation and cancer: a focus on AU-rich elements. Cell Mol. Life Sci..

[19] Stumpo DJ, Lai WS, Blackshear PJ (2010). Inflammation: cytokines and RNA-based regulation. Wiley Interdiscip. Rev. RNA.

[20] Alvarez E, Zhou W, Witta SE, Freed CR (2005). Characterization of the Bex gene family in humans, mice, and rats. Gene.

[21] Faria TN, LaRosa GJ, Wilen E, Liao J, Gudas LJ (1998). Characterization of genes which exhibit reduced expression during the retinoic acid-induced differentiation of F9 teratocarcinoma cells: involvement of cyclin D3 in RA-mediated growth arrest. Mol. Cell. Endocrinol..

[22] Quentmeier H (2005). Expression of BEX1 in acute myeloid leukemia with MLL rearrangements. Leukemia.

[23] Foltz G (2006). Genome-wide analysis of epigenetic silencing identifies BEX1 and BEX2 as candidate tumor suppressor genes in malignant glioma. Cancer Res..

[24] Kazi JU, Kabir NN, Ronnstrand L (2015). Brain-Expressed X-linked (BEX) proteins in human cancers. Biochim. Biophys. Acta..

[25] Koo JH, Smiley MA, Lovering RM, Margolis FL (2007). Bex1 knock out mice show altered skeletal muscle regeneration. Biochem. Biophys. Res. Commun..

[26] Vilar M (2006). Bex1, a novel interactor of the p75 neurotrophin receptor, links neurotrophin signaling to the cell cycle. EMBO J..

[27] Khazaei MR (2010). Bex1 is involved in the regeneration of axons after injury. J. Neurochem..

[28] Behrens M, Margolis JW, Margolis FL (2003). Identification of members of the Bex gene family as olfactory marker protein (OMP) binding partners. J. Neurochem..

[29] Jiang C, Wang JH, Yue F, Kuang S (2016). The brain expressed x-linked gene 1 (Bex1) regulates myoblast fusion. Dev. Biol..

[30] Aronow BJ (2001). Divergent transcriptional responses to independent genetic causes of cardiac hypertrophy. Physiol. Genomics.

[31] Murray EL, Schoenberg DR (2007). A + U-rich instability elements differentially activate 5’-3’ and 3’-5’ mRNA decay. Mol. Cell. Biol..

[32] Epelman S, Liu PP, Mann DL (2015). Role of innate and adaptive immune mechanisms in cardiac injury and repair. Nat. Rev. Immunol..

[33] Topkara VK (2011). Therapeutic targeting of innate immunity in the failing heart. J. Mol. Cell. Cardiol..

[34] Mann DL, Topkara VK, Evans S, Barger PM (2010). Innate immunity in the adult mammalian heart: for whom the cell tolls. Trans. Am. Clin. Climatol. Assoc..

[35] Mann DL (2011). The emerging role of innate immunity in the heart and vascular system: for whom the cell tolls. Circ. Res..

[36] Frantz S (1999). Toll4 (TLR4) expression in cardiac myocytes in normal and failing myocardium. J. Clin. Invest..

[37] Dubovy P, Jancalek R, Kubek T (2013). Role of inflammation and cytokines in peripheral nerve regeneration. Int. Rev. Neurobiol..

[38] Kyritsis N, Kizil C, Brand M (2014). Neuroinflammation and central nervous system regeneration in vertebrates. Trends Cell Biol..

[39] Beiter T (2015). Exercise, skeletal muscle and inflammation: ARE-binding proteins as key regulators in inflammatory and adaptive networks. Exerc. Immunol. Rev..

[40] Kharraz Y, Guerra J, Mann CJ, Serrano AL, Munoz-Canoves P (2013). Macrophage plasticity and the role of inflammation in skeletal muscle repair. Mediators Inflamm..

[41] Hanahan D, Weinberg RA (2011). Hallmarks of cancer: the next generation. Cell.

[42] Grivennikov SI, Greten FR, Karin M (2010). Immunity, inflammation, and cancer. Cell.

[43] Shalapour S, Karin M (2015). Immunity, inflammation, and cancer: an eternal fight between good and evil. J. Clin. Invest..

[44] Lee JK (2016). Oncogenic microtubule hyperacetylation through BEX4-mediated sirtuin 2 inhibition. Cell. Death Dis..

[45] St Johnston D (2005). Moving messages: the intracellular localization of mRNAs. Nat. Rev. Mol. Cell Biol..

[46] Soundararajan HC, Bullock SL (2014). The influence of dynein processivity control, MAPs, and microtubule ends on directional movement of a localising mRNA. eLife.

[47] Rahman N, Stewart G, Jones G (2010). A role for the atopy-associated gene PHF11 in T-cell activation and viability. Immunol. Cell Biol..

[48] Zhang Z (2011). DDX1, DDX21, and DHX36 helicases form a complex with the adaptor molecule TRIF to sense dsRNA in dendritic cells. Immunity.

[49] Soto-Rifo R, Rubilar PS, Ohlmann T (2013). The DEAD-box helicase DDX3 substitutes for the cap-binding protein eIF4E to promote compartmentalized translation initiation of the HIV-1 genomic RNA. Nucleic Acids Res..

[50] Ariumi Y (2014). Multiple functions of DDX3 RNA helicase in gene regulation, tumorigenesis, and viral infection. Front. Genet..

[51] Gu L, Fullam A, Brennan R, Schroder M (2013). Human DEAD box helicase 3 couples IkappaB kinase epsilon to interferon regulatory factor 3 activation. Mol. Cell Biol..

[52] Schroder M, Baran M, Bowie AG (2008). Viral targeting of DEAD box protein 3 reveals its role in TBK1/IKKepsilon-mediated IRF activation. EMBO J..

[53] Jia J, Arif A, Ray PS, Fox PL (2008). WHEP domains direct noncanonical function of glutamyl-Prolyl tRNA synthetase in translational control of gene expression. Mol. Cell.

[54] Mukhopadhyay R, Jia J, Arif A, Ray PS, Fox PL (2009). The GAIT system: a gatekeeper of inflammatory gene expression. Trends Biochem. Sci..

[55] Sanbe A (2003). Reengineering inducible cardiac-specific transgenesis with an attenuated myosin heavy chain promoter. Circ. Res..

[56] Haq S (2001). Differential activation of signal transduction pathways in human hearts with hypertrophy versus advanced heart failure. Circulation.

[57] Oka T (2006). Cardiac-specific deletion of Gata4 reveals its requirement for hypertrophy, compensation, and myocyte viability. Circ. Res..

[58] Wilkins BJ (2004). Calcineurin/NFAT coupling participates in pathological, but not physiological, cardiac hypertrophy. Circ. Res..

[59] Oka T, Dai YS, Molkentin JD (2005). Regulation of calcineurin through transcriptional induction of the calcineurin A beta promoter in vitro and in vivo. Mol. Cell. Biol..

[60] Liu Q, Busby JC, Molkentin JD (2009). Interaction between TAK1-TAB1-TAB2 and RCAN1-calcineurin defines a signalling nodal control point. Nat. Cell Biol..

[61] Shevchenko A, Wilm M, Vorm O, Mann M (1996). Mass spectrometric sequencing of proteins silver-stained polyacrylamide gels. Anal. Chem..

[62] Eismann T (2009). Peroxiredoxin-6 protects against mitochondrial dysfunction and liver injury during ischemia-reperfusion in mice. Am. J. Physiol. Gastrointest. Liver. Physiol..

[63] Kanisicak O (2016). Genetic lineage tracing defines myofibroblast origin and function in the injured heart. Nat. Commun..

