# Supplementary material for: BEX1 is an RNA-dependent mediator of cardiomyopathy
Source: Nat Commun. 2017 Nov 30;8:1875. doi: 10.1038/s41467-017-02005-1 (PMC5709413; doi:10.1038/s41467-017-02005-1)
Supplement: Supplementary file 1 — Supplementary Information [file 41467_2017_2005_MOESM1_ESM.pdf]

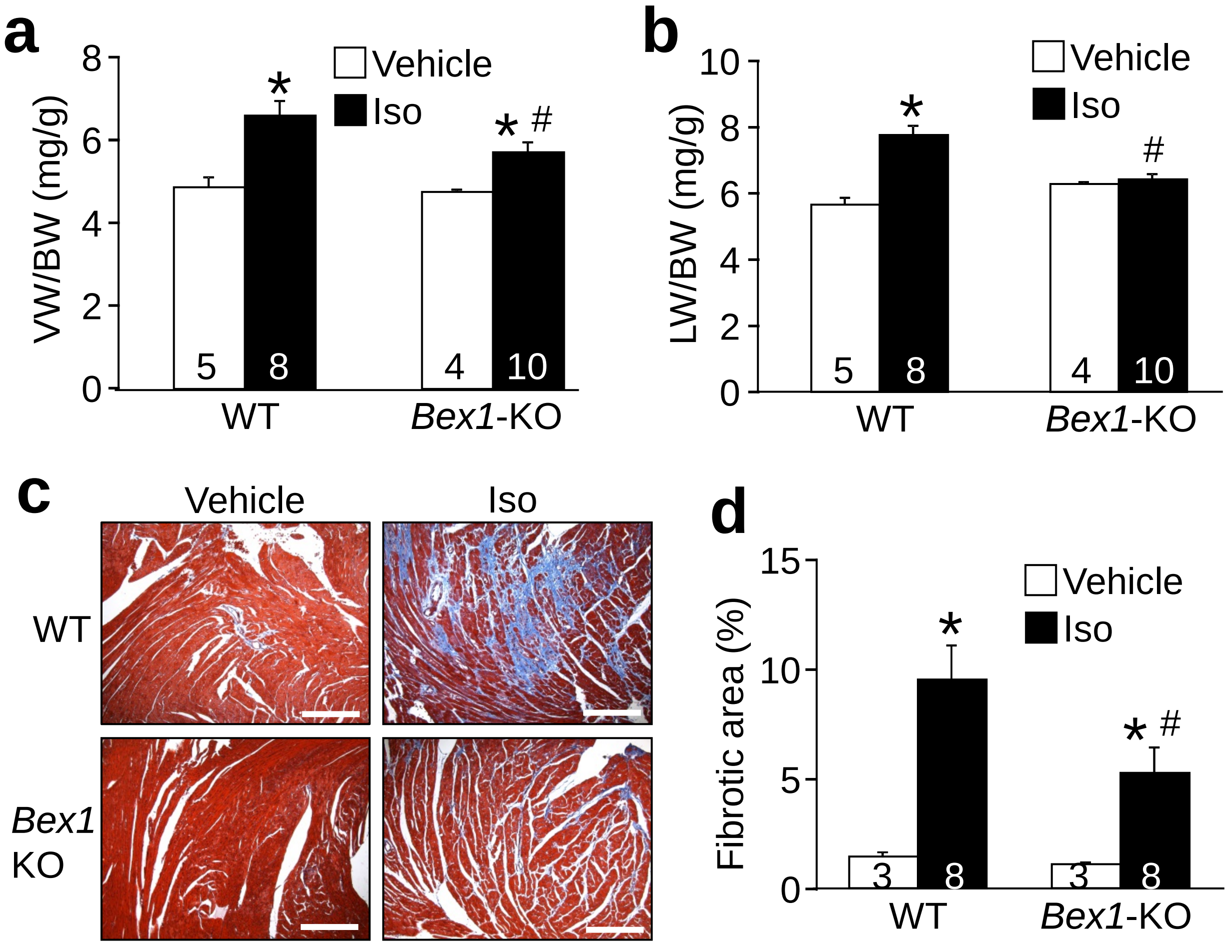

Supplementary Figure 1. (**a**), Ventricular weight to body weight (VW/BW) ratio from WT control and *Bex1*-knock out (*Bex1*-KO) mice subjected to 4-weeks of vehicle or isoproterenol infusion. (**b**), Lung weight to body weight (LW/BW) ratio from WT control and *Bex1*-KO mice subjected to 4-weeks of vehicle or isoproterenol infusion. (**c**), Representative Masson's trichrome-stained cardiac histological sections for fibrosis (**blue**) in mice of the indicated genotypes and treatments. Original magnification, x50 and scale bars are 200 µm. (**d**), Quantification of fibrosis from Masson's trichrome-stained sections of WT and *Bex1*-KO mice with vehicle or isoproterenol infusion using ImageJ software. *P<0.05 versus vehicle; #P<0.05 versus WT Iso. P values are 1-way ANOVA with Bonferroni correction. Number of animals used is shown in the bars in each panel. Error bars are s.e.m.

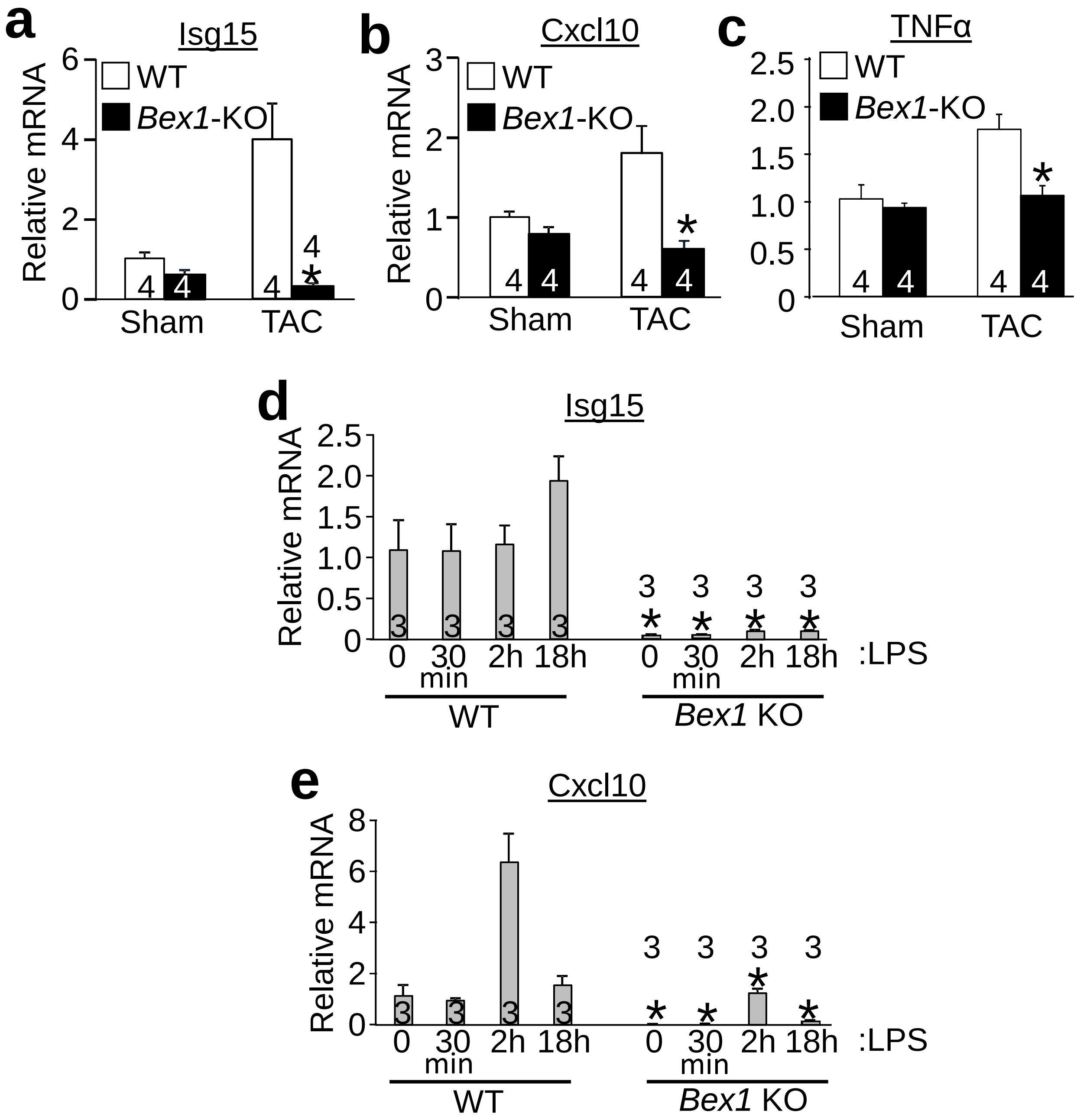

Supplementary Figure 2. (**a-c**) qPCR analysis of the indicated genes from hearts WT or *Bex1*-KO subjected to TAC or sham surgeries. *P<0.05 versus WT TAC. (**d,e**) qPCR analysis of the indicated genes from mouse embryonic fibroblasts (MEFs) WT or *Bex1*-KO with or without lipopolysaccharide (LPS) treatment for the indicated time. mRNA levels were normalized to Rpl7. *P<0.05 versus WT. P value is unpaired 2-tailed t-test. Sample numbers are shown in the graphs. Error bars are s.e.m.

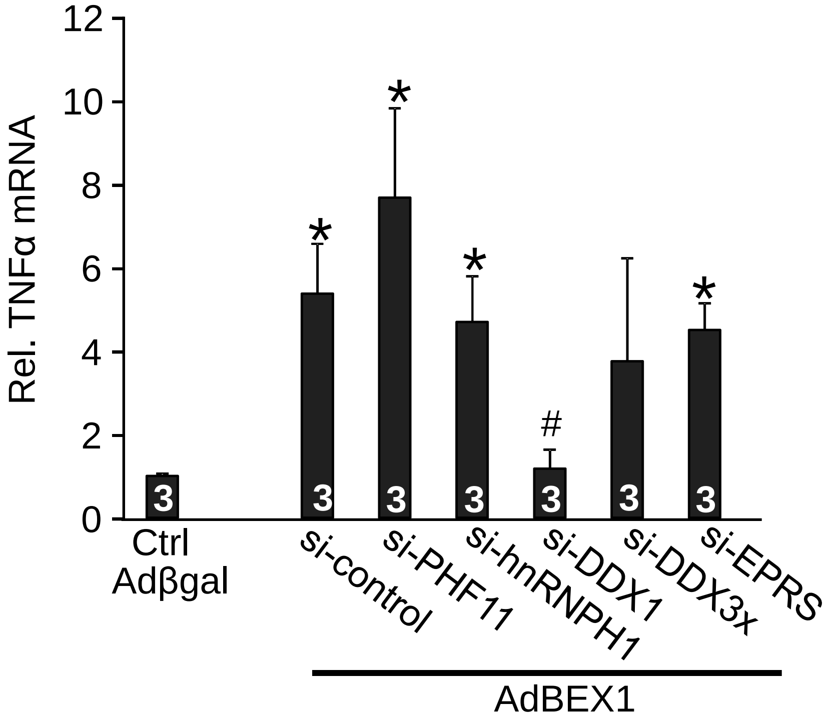

Supplementary Figure 3. qPCR analysis for TNFα mRNA levels from neonatal rat cardiomyocytes overexpressing BEX1 (AdBEX1) compared to β-galactosidase overexpressing control adenoviral infection (AdβGal). Treatments include control siRNAs or siRNAs targeting the indicated genes. mRNA levels were normalized to Rpl7. *P<0.05 versus Adβgal. #P<0.05 versus AdBEX1 si-control. P value is unpaired 2-tailed t-test. Sample numbers are shown in the bars of the graph. Error bars are s.e.m.

Fig 1b

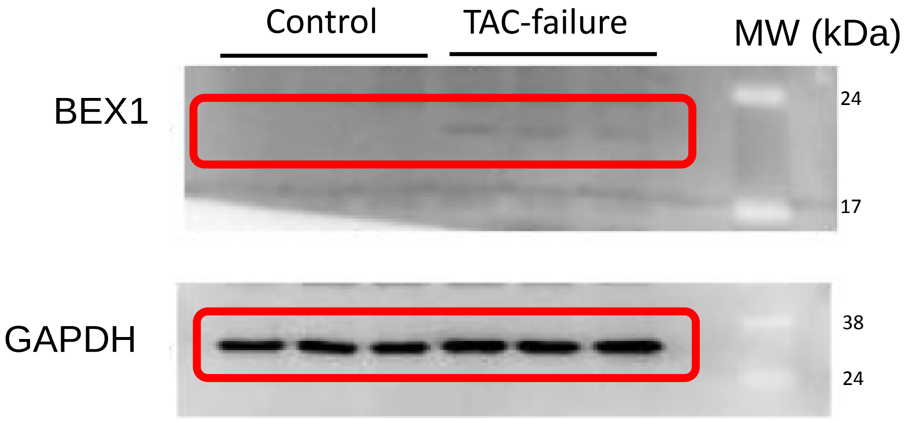

Supplementary Figure 4. Raw western blot gel images over the next 4 pages that refer to each of the designated images from the main figures

Fig 1d

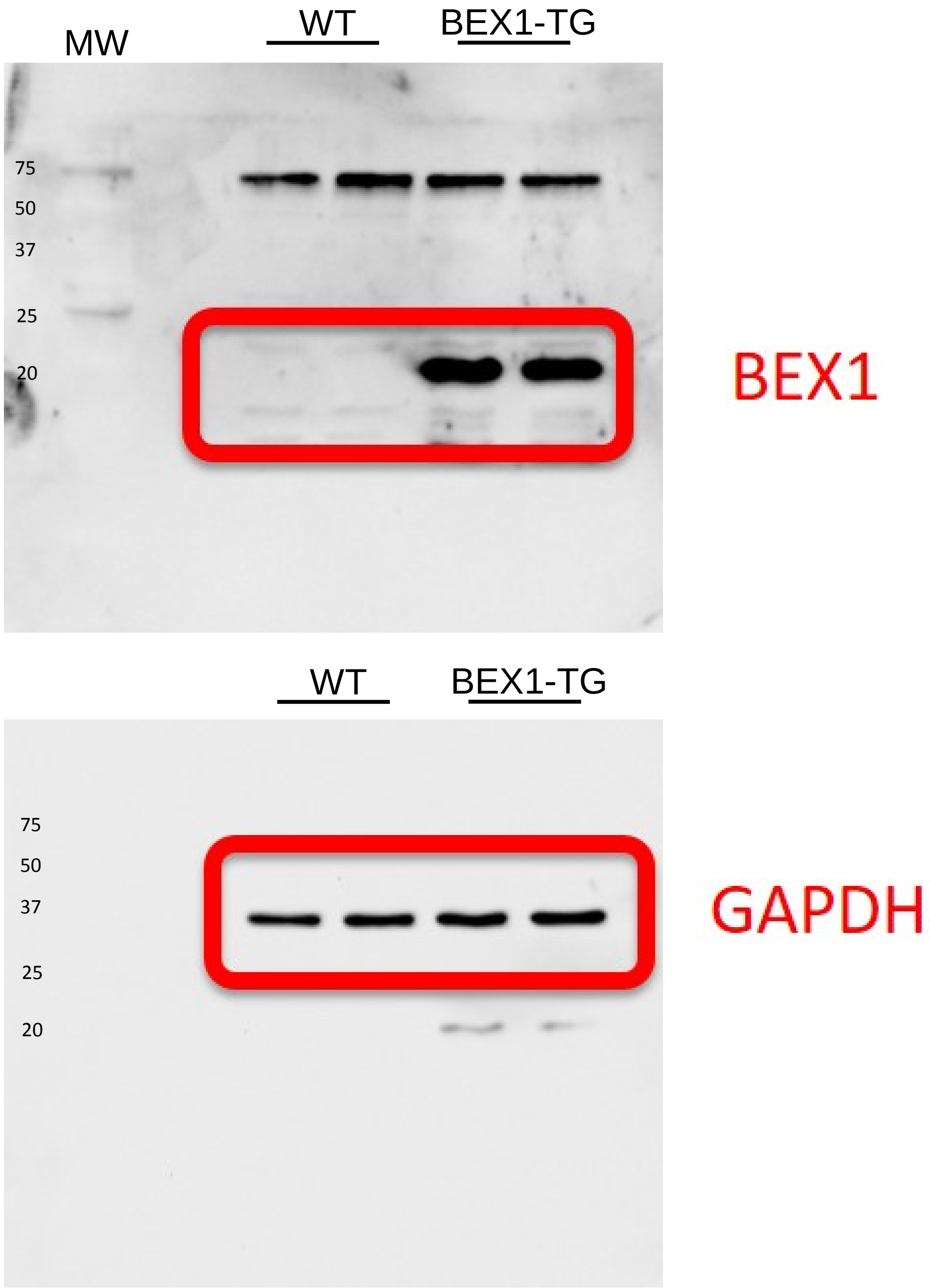

Fig 3d

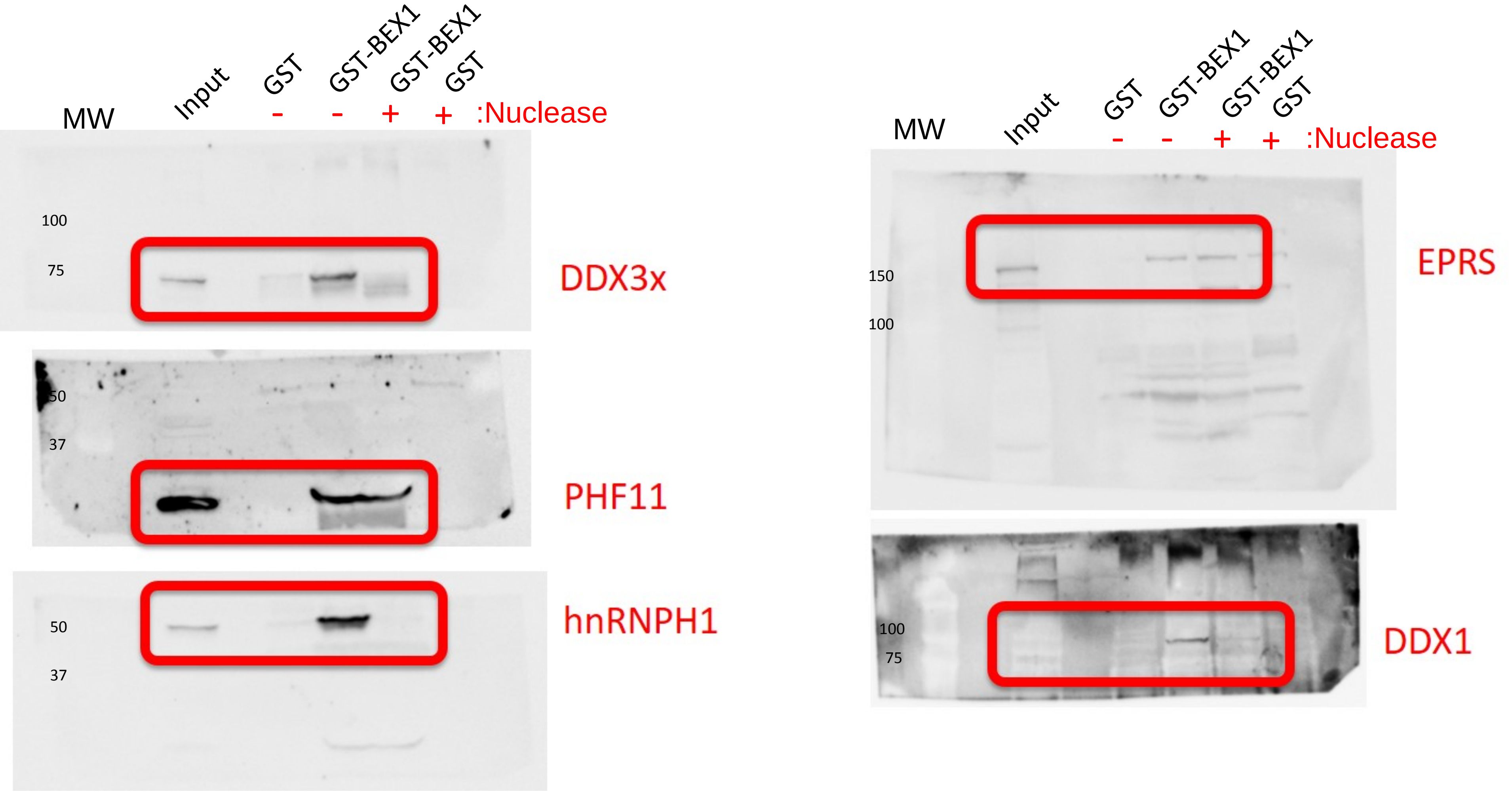
MW
Input
GST
GST-BEX1
GST-BEX1
GST
- - + + :Nuclease
100
75
DDX3x
50
37
PHF11
50
37
hnRNPH1
MW
Input
GST
GST-BEX1
GST-BEX1
GST
- - + + :Nuclease
150
100
EPRS
100
75
DDX1

Fig 3e

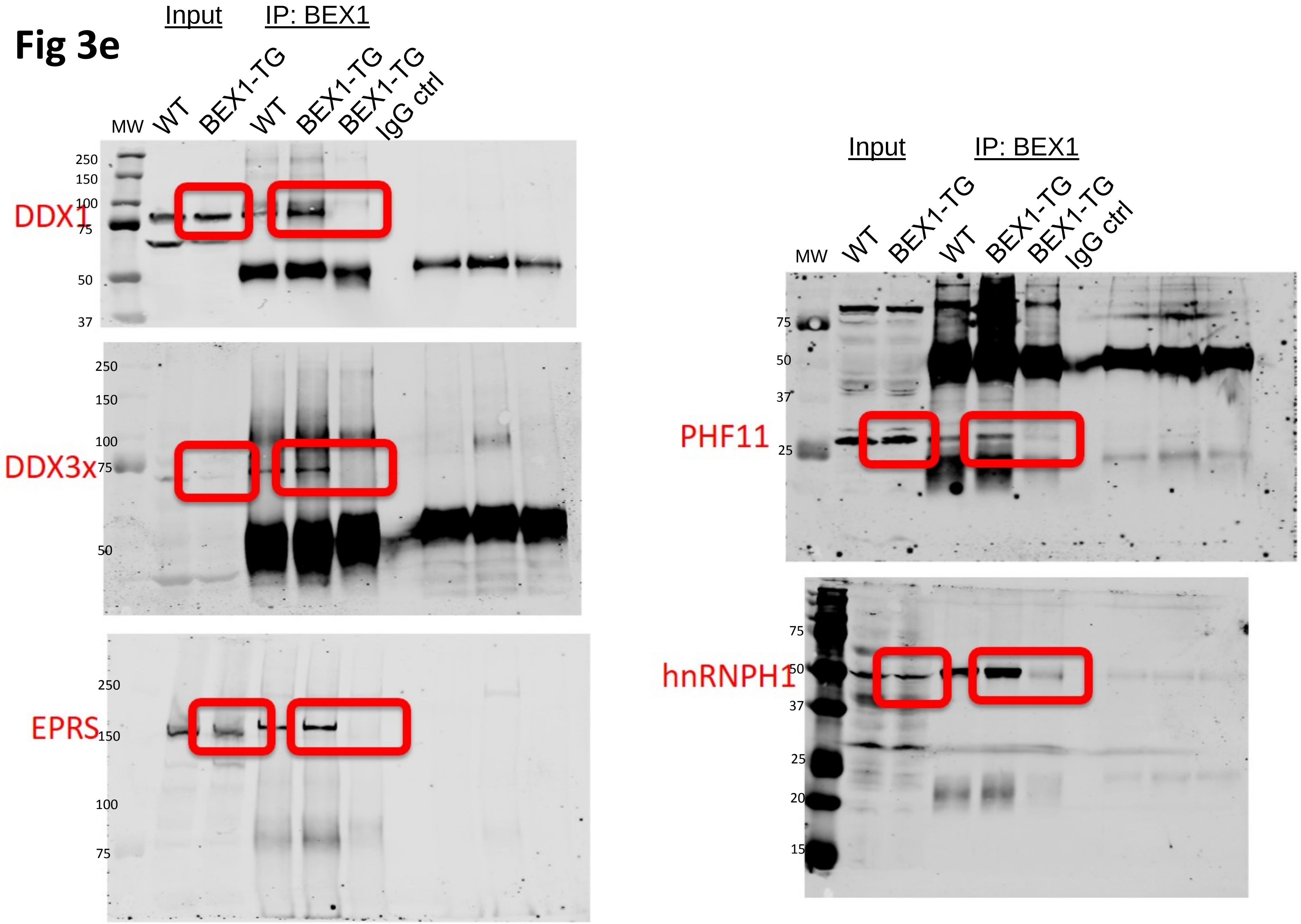
Input
IP: BEX1
MW
WT
BEX1-TG
WT
BEX1-TG
BEX1-TG
IgG ctrl
DDX1
250
150
100
75
50
37
DDX3x
250
150
100
75
50
EPRS
250
150
100
75
Input
IP: BEX1
MW
WT
BEX1-TG
WT
BEX1-TG
BEX1-TG
IgG ctrl
PHF11
75
50
37
25
hnRNPH1
75
50
37
25
20
15

Fig 3f

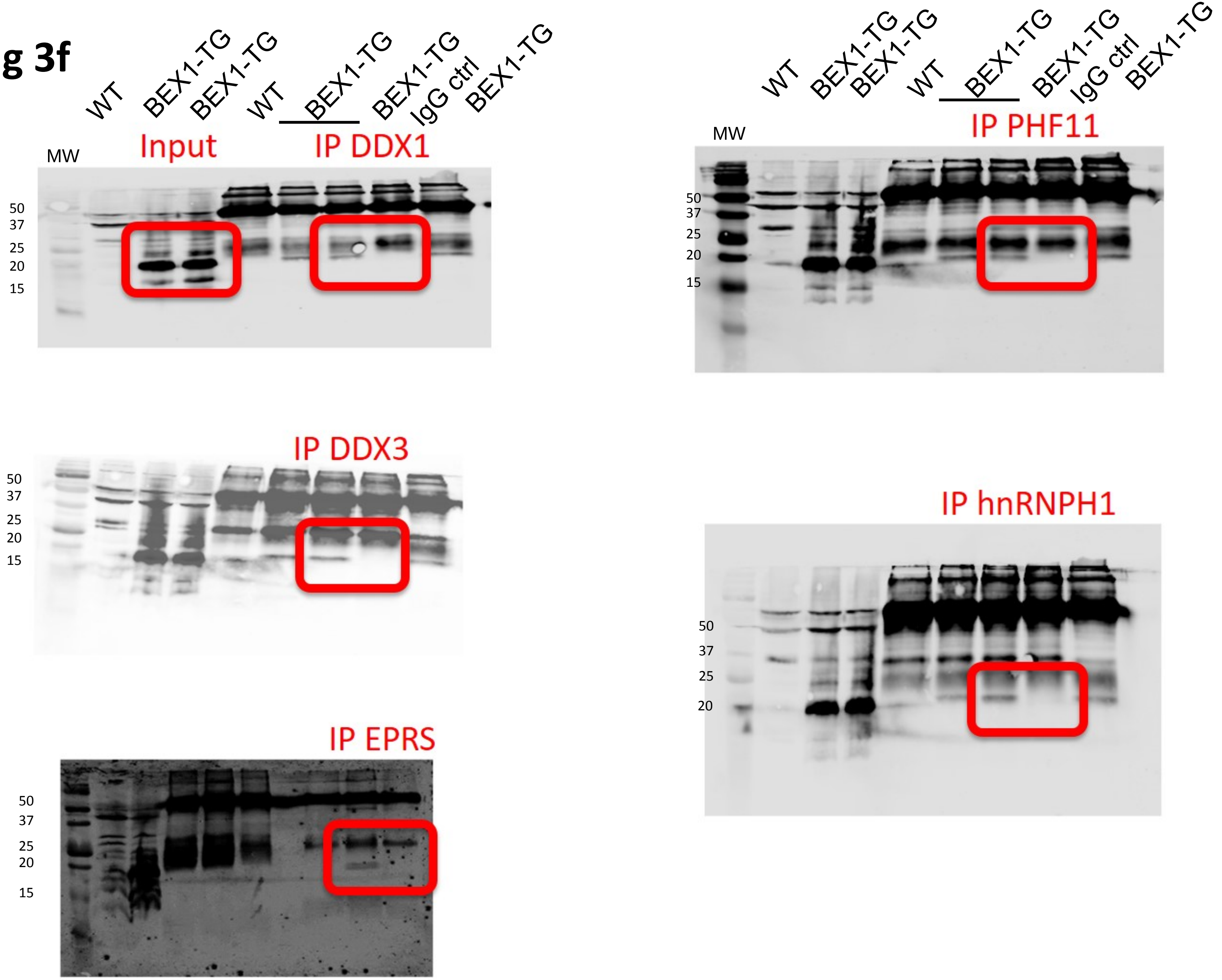
WT
BEX1-TG
BEX1-TG
WT
BEX1-TG
BEX1-TG
IgG ctrl
BEX1-TG
MW
Input
IP DDX1
50
37
25
20
15
MW
IP PHF11
50
37
25
20
15
IP DDX3
50
37
25
20
15
IP hnRNPH1
50
37
25
20
IP EPRS
50
37
25
20
15

Supplementary Table 1. Protein and Peptide Summaries from MASCOT Analysis of MALDI-TOF/TOF Data

Figure 3a, Band 1

1 **gi|163965357 Mass: 220364 Protein Score: 1188 Queries matched: 12**
nascent polypeptide-associated complex subunit alpha isoform a [Mus musculus]

Observed	Mr(expt)	Mr(calc)	ppm	Miss	Score	Expect	Rank	Peptide
1215.6207	1214.6134	1214.6619	-39.91	0	67	0.001	1	K.GVPISSALTQSR.L
1448.7987	1447.7914	1447.8511	-41.20	1	53	0.03	1	R.LSLNLKGPVSPPAR.N
1568.7687	1567.7614	1567.8206	-37.74	0	119	8e-09	1	K.VPVPAETQEVAVSSR.E
1836.9457	1835.9384	1836.0105	-39.26	0	38	1.1	1	K.GSNVALQPLVTQVPASQK.T
1943.9104	1942.9031	1942.9789	-38.98	0	90	6.9e-06	1	K.SVPFPDPPLAEISFSNAR.K
2134.0305	2133.0232	2133.0954	-33.81	0	121	5.6e-09	1	K.ETSAPSEGVTAVPLEIPPSPR.K
2148.0837	2147.0764	2147.1586	-38.28	1	132	4.3e-10	1	K.QVPAEILPSPQKTPEVTASR.L
2200.0444	2199.0371	2199.1172	-36.39	0	157	1.5e-12	1	K.ETPAPSPEGATTAPVQIPPSPR.K
2470.3154	2469.3081	2469.4067	-39.93	1	65	0.0022	1	K.NLPISALVNVGAPVSPAQAGLPTRK.D
2630.3105	2629.3032	2629.3963	-35.39	1	143	3.9e-11	1	K.TAVPKETSAPSEGVTAVPLEIPPSPR.K
2668.3013	2667.2940	2667.3868	-34.77	1	102	4.5e-07	1	K.TAAPKETPAPSPEGATTAPVQIPPSPR.K
2710.2869	2709.2796	2709.3756	-35.43	1	109	9.8e-08	1	K.TSTSQVPSQGTLNLKGTAPCPPDVVR.A + Carbamidomethyl (C)

Figure 3a, Band 2

1 **gi|223461182 Mass: 169856 Protein Score: 437 Queries matched: 8**
Glutamyl-prolyl-tRNA synthetase [Mus musculus]

Observed	Mr(expt)	Mr(calc)	ppm	Miss	Score	Expect	Rank	Peptide
1064.5156	1063.5083	1063.5410	-30.77	0	28	8.2	1	K.QFIAAQGSSR.S
1070.5920	1069.5847	1069.6244	-37.07	1	57	0.0091	1	K.KGDIIQLQR.R
1178.6497	1177.6424	1177.6931	-43.07	1	42	0.32	1	K.GVPVRLEVGPR.D
1188.5525	1187.5452	1187.5870	-35.14	0	28	9.8	1	R.LNQWCNVVR.W + Carbamidomethyl (C)
1400.7545	1399.7472	1399.8075	-43.05	0	63	0.0026	1	R.VYEELLAIPVVR.G
1460.7506	1459.7433	1459.8035	-41.20	0	69	0.00075	1	K.VLEDIQLNLFTR.A
1650.7954	1649.7881	1649.8625	-45.05	0	56	0.016	1	R.VSETVAFTDVNSILR.Y
1963.8547	1962.8474	1962.9323	-43.25	0	91	4.8e-06	1	K.FAGGDYTTTIEAFISASGR.A

Figure 3a, Band 3

1 **gi|199025 Mass: 117603 Protein Score: 135 Queries matched: 3**
microtubule-associated protein 4 [Mus musculus]

Observed	Mr(expt)	Mr(calc)	ppm	Miss	Score	Expect	Rank	Peptide
1447.6920	1446.6847	1446.7314	-32.29	0	48	0.087	1	K.ATSPSTLVSTGPSSR.S
1536.7343	1535.7270	1535.7805	-34.79	1	37	1.2	1	K.VGSTENIKHQPGGGR.A
1630.7319	1629.7246	1629.7781	-32.80	0	52	0.043	1	R.NTTPTGAAPPAGMTSTR.V

Figure 3b, Band 1

1 **gi|19527256 Mass: 82448 Protein Score: 91 Queries matched: 2**
ATP-dependent RNA helicase DDX1 [Mus musculus]

Observed	Mr(expt)	Mr(calc)	ppm	Miss	Score	Expect	Rank	Peptide
1111.6276	1110.6203	1110.6761	-50.19	0	38	0.54	1	R.ELLIIGGVAAR.D
1165.5530	1164.5457	1164.5961	-43.28	0	54	0.017	1	R.FLICTDVAAR.G + Carbamidomethyl (C)

Fig 3b, Band 2

1 **gi|309319 Mass: 70793 Protein Score: 400 Queries matched: 5**
heat shock protein 70 cognate [Mus musculus]

Observed	Mr(expt)	Mr(calc)	ppm	Miss	Score	Expect	Rank	Peptide
1180.5731	1179.5658	1179.6135	-40.45	1	35	1.1	1	K.VQVEYKGETK.S
1199.6302	1198.6229	1198.6670	-36.75	0	85	1.2e-05	1	K.DAGTIAGLNVLR.I
1487.6500	1486.6427	1486.6940	-34.49	0	101	3.2e-07	1	R.TTPSYVAFTDTER.L
1627.8483	1626.8410	1626.9053	-39.51	1	88	8.1e-06	1	R.QATKDAGTIAGLNVLR.I
1981.9078	1980.9005	1980.9905	-45.43	0	67	0.00099	1	K.TVTNAVVTVPAYFNDSQR.Q

2 **gi|6753620 Mass: 73056 Protein Score: 103 Queries matched: 2**
ATP-dependent RNA helicase DDX3X [Mus musculus]

Observed	Mr(expt)	Mr(calc)	ppm	Miss	Score	Expect	Rank	Peptide
1168.6567	1167.6494	1167.6975	-41.21	0	47	0.067	1	K.SPILVATAVAAR.G
1524.7208	1523.7135	1523.7732	-39.19	0	59	0.0058	1	R.VGNLGLATSFFNER.N
