# Supplementary material for: BEX1 is an RNA-dependent mediator of cardiomyopathy
Source: Nat Commun. 2017 Nov 30;8:1875. doi: 10.1038/s41467-017-02005-1 (PMC5709413; doi:10.1038/s41467-017-02005-1)
Supplement: Supplementary file 2 — Description of Additional Supplementary Files [file 41467_2017_2005_MOESM2_ESM.pdf]

Description of Additional Supplementary Files

File Name: Supplementary Data 1
Description: Complete list of RIPseq for BEX1-RNA interaction
